# Academic Entitlement in Osteopathic Medical Students

**DOI:** 10.7759/cureus.88859

**Published:** 2025-07-27

**Authors:** Sidney Brown, Abigail Rodgers, Danielle Fastring

**Affiliations:** 1 Medical School, William Carey University College of Osteopathic Medicine, Hattiesburg, USA; 2 Student Research and Preclinical Sciences, William Carey University College of Osteopathic Medicine, Hattiesburg, USA

**Keywords:** academic entitlement, entitled expectations, osteopathic medical students, osteopathic schools, personal responsibility

## Abstract

Introduction: Academic entitlement (AE) is the tendency to hold a certain expectation of academic success without taking personal responsibility. The purpose of this study was to define AE in osteopathic medical education and assess AE in osteopathic medical students.

Methods: A cross-sectional survey (n=145) was conducted at a school of osteopathic medicine using the Qualtrics Survey Platform (Provo, Utah, United States). The survey consisted of demographic data, academic performance, student satisfaction, and responses from two validated AE instruments. To be eligible, students had to be enrolled full time and a member of the OMS-1 to OMS-4 cohorts.

Results: Students classified as academically entitled were significantly more likely to agree with statements reflecting externalized responsibility (ER) and entitled expectations (EE) (p<0.05). Additionally, students with higher AE scores were 3.8 times more likely to have a GPA below 85% (p=0.012), indicating a possible relationship between AE and lower academic success.

Conclusion: Identifying academically entitled students early in their academic career provides an opportunity to intervene in the areas of unrealistic expectations, negative peer dynamics, and unprofessional conduct.

## Introduction

Academic entitlement (AE) is a tendency to hold a certain expectation of academic success without taking personal responsibility [1]. AE is an emerging concern in higher education and poses particular risks in medical education and training. Such attitudes undermine the doctrines of the Osteopathic Oath, especially the statement "I will look with respect and esteem upon all those who have taught me my art". Professionalism is an essential pillar of medical training and crucial for fostering trust and delivering high-quality patient care. Yet, entitled attitudes hinder the development of empathy, humility, and responsibility, which are vital for a successful career in healthcare [2]. In healthcare, where patient well-being relies on accountability, AE can undermine the development of professionalism and ethical responsibility [3]. Although concerning, AE is not unchangeable; targeted interventions, including clear expectations and constructive feedback, early in the educational journey can positively influence student attitudes [4]. Addressing entitlement early in medical education enables educators to foster a more responsible and self-reliant approach to learning among students. Proactively identifying and mitigating AE in osteopathic medical students is essential to cultivating dependable, professional clinicians equipped for the demands of patient care.

In various academic literature, there is a notion of occasional animosity between the student and professor within institutions of higher education, but this incivility may extend beyond the primary relationship [5]. It can change the dynamic of group projects and impact essay writing and research and other professional and academic domains [6]. The root of the negative behavior is often a sense of AE held by the student. A study at the University of Oklahoma by Chowning and Campbell [7] found a connection between higher AE and an external locus of control with traits of disagreeableness and disregard for others, and conversely, having an internal locus of control was found to be a stronger predictor of academic achievement. A four-part study by the same researchers found that students with higher external responsibility poorly evaluated experimenters, which may similarly affect the course evaluations distributed to osteopathic medical students in their courses [7].

Previous research in pharmacy and dental students found that AE correlates with lower academic performance, raising concerns about its implications for clinical competency in medical trainees [7,8]. In one study, graduating pharmacy students with lower levels of AE required fewer reassessments and remediations, demonstrating a clear inverse relationship between AE and academic performance [1]. At the 2024 American Dental Education Association Annual Session, four panelists discussed a survey project assessing AE in dental students across five US dental schools (n=502) using validated instruments from Chowning and Campbell and Kopp et al. [7,8]. Findings indicated that students who scored above the median AE score identified as "more academically entitled", with 13.9% of the respondents falling into this category for either one or both survey instruments. Although the data did not find a statistically significant difference, there was an association between low AE and a high degree of academic performance [9].

While AE has been examined in undergraduate and graduate students, its prevalence in osteopathic medical education remains unexplored. Osteopathic training emphasizes holistic, patient-centered care, making entitlement attitudes particularly concerning. Additionally, medical education's hierarchical nature may create tension between student expectations and faculty standards, exacerbating entitlement. This study aims to fill this gap by assessing AE in osteopathic students and identifying potential intervention points. We hypothesize that higher AE scores would correlate with lower academic success (GPA <85%) and that entitled students would demonstrate significant differences in AE subdomains compared to non-entitled peers.

## Materials and methods

This cross-sectional, observational study was conducted at a school of osteopathic medicine in 2024 and was approved by the Institutional Review Board (IRB) of William Carey University (approval number: 2023-040). An anonymous, self-administered survey was developed using two externally validated instruments that assess AE. The full survey is accessible in the Appendices. Instrument A captures the subdomains of externalized responsibility (ER) and entitled expectations (EE), while Instrument B assesses student attitudes toward grading practices and perceived academic responsibility. These instruments have previously demonstrated strong internal consistency in studies of pharmacy and dental students [7,8].

The survey was distributed via the Qualtrics platform (Provo, Utah, United States) to all actively enrolled osteopathic medical students (OMS-I through OMS-IV). Recruitment occurred through institutional email, and the survey remained open for two months. To be eligible, participants had to be enrolled full time and provide informed consent. Responses with missing or duplicate entries were excluded, yielding a final sample size of 145.

Participants self-reported demographic information and GPA and were stratified into categories based on anticipated graduation year and academic performance. Though student success in osteopathic programs is not merely based on GPA, for the purposes of this study, students with a GPA ≥85% were classified as academically successful, while those with a GPA <85% were classified as academically unsuccessful. This threshold was determined post hoc through exploratory data analysis to ensure balanced group sizes and is not part of a validated performance scale. Responses to AE statements were rated using a 5-point Likert scale ranging from "strongly disagree" to "strongly agree". Negatively worded items were reverse-coded to ensure that higher scores reflected greater AE. Composite AE scores were calculated by summing responses across all items from both instruments. Participants were categorized as "entitled" if their combined AE score exceeded the group median (cutoff: 48 points). 

Descriptive statistics (median and interquartile range) were calculated for all survey items. Associations between entitlement status and demographic or academic variables were assessed using chi-squared tests. Chi-squared tests were also used to compare Likert response distributions between entitled and non-entitled groups. Continuity corrections were applied where appropriate, and Fisher's exact test was used in 2×2 tables with small expected cell counts. A two-tailed p-value of <0.05 was considered statistically significant. All analyses were conducted using IBM SPSS Statistics for Windows, Version 28.0 (IBM Corp., Armonk, New York, United States).

## Results

Demographic information from participants (n=145) can be found in Table 1. More than half of respondents identified as female (57.2%), and the majority (n=93; 64.1%) were aged between 26 and 40. The highest response rate was among OMS-1 students (Class of 2027; n=41; 28.3%). Of the total respondents, 32 students (64%) were categorized as academically successful, with a GPA greater than 85%.

**Table 1:** Survey respondent demographic data and academic performance (n=145) OMS refers to osteopathic medical student. The numeric suffix denotes the academic year (e.g., OMS-1 indicates a first-year osteopathic medical student, OMS-2 a second-year, and so on).

Characteristic	n (%)
Gender	
Male	55 (37.9)
Female	83 (57.2)
Non-binary	3 (2.1)
Transgender male	1 (0.7)
Transgender female	2 (1.4)
Prefer not to say	1 (0.7)
Age group (years)	
18-25	51 (35.2)
26-40	93 (64.1)
>40	1 (0.7)
Graduating year	
2024	39 (26.9)
2025	27 (18.6)
2026	38 (26.2)
2027	41 (28.3)
Academic success	
Successful (GPA >85%)	32 (64%)
Reassessments in OMS-1	
One	18 (54.5)
Two	6 (18.2)
Three or more	9 (27.3)
Reassessments in OMS-2	
One	16 (44.4)
Two	11 (30.6)
Three or more	9 (25)
Reassessments in OMS-3	
One	1 (0.7)
Two	0 (0)
Three or more	1 (0.7)
Course remediation	
OMS-1 (n=105)	5 (4.8)
OMS-2 (n=92)	5 (5.4)
OMS-3 (n=76)	2 (2.6)
Entitled	
Yes (combined A+B score >48)	41 (45.1)

The median Likert scale response for each item on the Academic Entitlement Instruments by category of entitlement is presented in Table 2. Because the Likert scale data were non-normally distributed, median comparisons were conducted using the Mann-Whitney U test. Significant differences in responses between those categorized as entitled and not entitled were found for all statements. Responses for statements in Instrument A (externalized responsibility) showed that entitled students more frequently agreed with statements such as "My professors are obligated to help me prepare for exams" (median score of 4.0) compared to non-entitled students (median score of 3.0) (p=0.003), with a Mann-Whitney U p-value of 0.003. Similar trends were seen in Instrument B, where entitled students agreed with the statement "It is the professor's responsibility to make it easy for me to succeed" (median=4.0 vs. 3.0; p=0.025 by Mann-Whitney U). A significant association was found between AE and GPA. Among students classified as academically successful (GPA >85%), 64% were not entitled and 36% were entitled (p=0.012) (Table 3). No significant difference was found between those who were entitled and those who weren't with regard to reassessment, course remediation, or first quartile of class rank (Table 3).

**Table 2:** Differences in Academic Entitlement Instrument statements EE: entitled expectations; ER: externalized responsibility *p<0.05. P-values are two-tailed and considered significant at p<0.05. Chi-squared test used for all comparisons (df=1 unless otherwise noted). Fisher's exact test was used where expected cell counts were small. Values are presented as median (interquartile range (IQR)) based on 5-point Likert responses.

Category	Statement	Not entitled	Entitled	Chi-square (df=1)	P-value*
		Median (SD)	Median (SD)		
EE	My professors are obligated to help me prepare for exams.	3 (4.0)	4 (4.0)	10.69	0.003
ER	Professors are employees who get money for teaching.	2 (3.0)	2 (4.0)	5.06	0.042
EE	Professors must be entertaining to be good.	2 (4.0)	3 (4.0)	14.97	<0.001
EE	I should never receive a zero on an assignment that I turned in.	2 (4.0)	3 (4.0)	12.88	<0.001
EE	My professors should curve my grade if I am close to the next letter grade.	2 (4.0)	3 (4.0)	18.14	<0.001
ER	I believe that the university does not provide me with the resources I need to succeed.	2 (4.0)	3 (4.0)	9.77	0.004
ER	For group work, I should receive the same grade as the other group members regardless of my level of effort.	1.5 (4.0)	2 (3.0)	0.25	0.804
EE	My professors should reconsider my grade if I am close to the grade I want.	2 (4.0)	3 (4.0)	9.78	0.004
ER	I believe that it is my responsibility to seek out the resources to succeed.	2 (2.0)	2 (3.0)	15.84	<0.001
ER	Most professors do not really know what they are talking about.	2 (3.0)	2 (4.0)	5.15	0.043
ER	It is unnecessary for me to participate in class when the professor is paid for teaching, not for asking questions.	2 (2.0)	3 (4.0)	9.78	0.004
ER	I am not motivated to put a lot of effort into group work, because another group member will end up doing it.	1 (1.0)	2 (3.0)	14.53	<0.001
ER	If I do poorly in a course and I could not make my professor's office hours, the fault lies with my professor.	1 (2.0)	2 (3.0)	26.35	<0.001
ER	For group assignments, it is acceptable to take a back seat and let others do most of the work if I am busy.	1 (3.0)	2 (2.0)	14.46	<0.001
ER	If I miss class, it is my responsibility to get the notes.	1 (4.0)	2 (4.0)	19.87	<0.001
-	If I am struggling in a class, the professor should approach me and offer to help.	2 (3.0)	3 (4.0)	14.34	<0.001
-	Professors should only lecture on material covered in the textbook and assigned readings.	2 (3.0)	3 (4.0)	17.78	<0.001
-	I should be given the opportunity to make up a test, regardless of the reason for the absence.	2 (4.0)	3 (4.0)	8.54	0.003
-	If I don't do well on a test, the professor should make tests easier or curve grades.	1.5 (3.0)	2 (4.0)	10.27	0.001
-	It is the professor's responsibility to make it easy for me to succeed.	2 (3.0)	2 (4.0)	4.99	0.025
-	If I cannot learn the material for a class from lecture alone, then it is the professor's fault when I fail the test.	2 (4.0)	3 (4.0)	27.97	<0.001
-	I am a product of my environment. Therefore, if I do poorly in class, it is not my fault.	1 (2.0)	2 (2.0)	12.13	0.002
-	Because I pay tuition, I deserve passing grades.	1 (1.0)	2 (2.0)	11.24	0.002

**Table 3:** Comparison of student characteristics and academic performance of more entitled and less entitled students for Academic Entitlement Instruments A and B OMS refers to osteopathic medical student. The numeric suffix denotes the academic year (e.g., OMS-1 indicates a first-year osteopathic medical student, OMS-2 a second-year, and so on). *p<0.05. Chi-squared test used for all comparisons (df=1 unless otherwise noted). P-values are two-tailed and considered significant at p<0.05. Fisher's exact test was used where expected cell counts were small. Values in parentheses indicate percentages.

	Not entitled	Entitled	Chi-squared test statistic (df=1)	P-value*
Reassessments in OMS-1				
One	6 (33.3)	12 (66.7)	0.64	0.726
Two	1 (25)	3 (75)		
Three or more	1 (16.7)	5 (83.3)		
Reassessments in OMS-2				
One	9 (60)	6 (40)	0.57	0.752
Two	4 (44.4)	5 (55.6)		
Three or more	4 (57.1)	3 (42.9)		
Reassessments in OMS-3				
One	1 (100)	0 (0)	0.83	0.363
Course remediation				
OMS-1	2 (66.7)	1 (33.3)	0.17	0.681
OMS-2	2 (66.7)	1 (33.3)	0.19	0.667
OMS-3	1 (100)	0 (0)	0.71	0.398
First quartile of class rank	17 (65.4)	9 (34.6)	0.85	0.356
GPA: academically successful (GPA >85%)	32 (64)	18 (36)	6.37	0.012

There was not a significant difference in AE among those who had completed one, two, or three reassessments or those who had completed course remediations across all cohort years (OMS-1 to OMS-4) (Table 3). In terms of quartile rank, there was not a statistically significant difference in rank between those who were entitled and those who were not (p=0.346). This suggests that while AE correlates with lower GPA, it does not predict formal academic interventions such as remediation. Academically unsuccessful students (GPA <85%) were 3.8 times more likely to exhibit AE (p=0.012) (Table 3).

## Discussion

The survey instruments used in this study have demonstrated internal consistency in prior studies with pharmacy and dental students, but they have not been formally validated in osteopathic medical students. As such, these findings should be interpreted as exploratory, and future studies should conduct psychometric validation (e.g., confirmatory factor analysis, test-retest reliability) to strengthen reliability in this population. While participants were categorized as "entitled" based on scoring above the median AE score (cutoff: 48 points), this threshold was determined for exploratory comparison and is not a validated classification standard. Furthermore, the 85% GPA threshold was selected based on exploratory data analysis to maintain balanced group sizes. However, future research should assess whether alternative classification methods, such as quartiles or standardized z-scores, yield similar trends. Additionally, grading policies may vary across osteopathic institutions, potentially influencing the applicability of these results. 

Academic entitlement and academic performance

There was not a significant difference in the percentage of participants in the first quartile for class rank between those who were "entitled" and those who were "not entitled". There were only 26 students who were in the first quartile of class rank. Though 65.4% within the first quartile of class rank were classified as "not entitled" and 36% were classified as "entitled", this difference did not reach statistical significance. This may be largely due to sample size and a lack of power to detect a true effect, should one exist (type II error).

In contrast to class rank, this study did find a significant association between AE and lower academic success (GPA <85). Students with higher AE scores were 3.8 times more likely to fall below this threshold. This aligns with prior research showing that students with lower GPAs tend to externalize responsibility (Instrument A) and hold entitled expectations (Instrument B). However, causality cannot be established. While AE may contribute to lower academic success, it is also possible that students struggling academically develop entitlement as a coping mechanism, particularly if they feel unsupported or unfairly assessed. Future studies should explore longitudinal relationships between AE and academic outcomes to determine whether AE precedes or results from academic difficulties.

These findings highlight a pattern of entitlement correlating with lower academic performance and externalized responsibility. This phenomenon corresponds with harboring unrealistic expectations within academia, adversely affecting peer dynamics, and displaying unprofessional conduct in their medical endeavors. The findings emphasize the importance of addressing entitlement not only to improve academic outcomes but also to prevent potential negative impacts on professionalism and patient care. Entitled attitudes are capable of hindering the development of essential qualities like empathy and responsibility, which are foundational to effective patient-centered care.

While acknowledging the potential for recall bias, we urge faculty at osteopathic medical institutions to remain vigilant regarding potential AE among students. Such individuals often exhibit an external locus of control and heightened anxiety levels, thereby impeding the learning process [10]. Early identification serves as a crucial step toward fostering a more supportive educational environment that nurtures the professional growth of future osteopathic physicians.

The impact of AE in osteopathic education parallels findings from studies conducted with pharmacy students [11]. At the graduate level, AE may contribute to grade inflation, whether intentional or not, raising significant concerns about maintaining academic standards [12]. Additionally, AE fosters student incivility, manifesting as unprofessional behaviors such as disregarding established professional hierarchies, avoiding necessary communication, arriving late, and other lapses in professionalism [11].

From a faculty perspective, repeated encounters with consumer-minded students and classroom incivility foster pessimism among the faculty, and if incivility continues unchecked, students feel bold enough to escalate disruptive behaviors [5]. This is challenging for junior faculty, who may lack the confidence or authority to manage such behaviors, but even experienced faculty's morale is diminished over time [11]. Administrators and faculty members need to be aware of these dynamics and offer support to colleagues affected by AE [11]. Examples of ways to mitigate these issues include clearly communicating academic and professional standards, establishing transparent policies on behavior and performance, and incorporating student self-reflection assignments and case-based scenarios to highlight the consequences of entitlement [4].

Entitled students often rely on peers to carry their workload, which not only strains team relationships but also reduces the potential for meaningful learning [13]. This sense of entitlement can also make it challenging for these students to accept roles that require them to follow rather than lead, which limits their ability to integrate smoothly into the hierarchical structure of healthcare teams [13]. Expanding on the mitigation of AE, collaborative reflective practice, which involves engaging students in activities where they reflect on their own attitudes and behaviors and consider the perspectives and experiences of their peers, provides a means of promoting self-awareness, empathy, and a sense of shared responsibility [13]. This provides a new perspective of addressing student attitudes.

Now, there is a disparity between faculty and student perspectives regarding what constitutes inappropriate behavior in the classroom; faculty often view actions such as the use of technology during lectures or leaving class without permission as disruptive, whereas students are generally more lenient and less likely to see these behaviors as problematic. This misalignment of expectations creates challenges for faculty in maintaining classroom discipline and upholding academic standards, which can lead to a decline in faculty morale over time [14].

Moreover, female faculty members are disproportionately affected by incivility. Students often challenge their authority more than their male counterparts, which is compounded by students' casual treatment of female professors. Such behavior undermines the professional respect faculty deserve and places additional stress on those already navigating the challenges of maintaining classroom order [5]. The burden of incivility is particularly acute for instructors who are younger and female, hold lower academic rank, or belong to underrepresented minority groups. Additionally, faculty teaching at public institutions, at research-intensive universities, or in large lecture-based settings are also significantly impacted [5]. These patterns suggest that AE may reinforce or exacerbate existing structural inequities within academic institutions, particularly those related to gender, race, age, and academic rank, by contributing to differential treatment of faculty based on perceived authority. Recognizing these challenges faced by faculty is important as they navigate efforts to reduce AE.

It is important to highlight that the medical students' academically entitled attitudes are not permanent. Medical students' attitudes may change as they gain exposure to the demands of the clinical environment [15]. The transition from an academic setting to patient-centered practice encourages the development of accountability and resilience, and this may counteract entitled attitudes cultivated earlier in their education. This strikes the importance of early identification and intervention strategies to help students adapt to the realities of patient care and professional responsibility. By proactively addressing entitlement early on, medical educators can foster a more supportive environment that aids longitudinal development into competent and empathetic professionals.

## Conclusions

Overall, this study highlights a significant association between AE and lower academic success among osteopathic medical students. Students with GPAs below 85% were more likely to exhibit entitled attitudes, particularly externalized responsibility and unrealistic academic expectations. Early identification of students with these traits is crucial, as it may enable timely interventions that promote personal accountability, strengthen peer collaboration, and reinforce professionalism within osteopathic training. Multifactorial strategies to mitigate AE could include increased administrative support for faculty, adjustments to instructor evaluation practices, revisions to admissions protocols, and enhanced student accountability measures.

Future research should explore longitudinal changes in AE as students progress through medical school, as well as assess the effectiveness of faculty-led interventions aimed at reducing entitlement. Additionally, studies should evaluate the long-term impact of AE mitigation strategies on student development and clinical performance. Although the instruments used in this study have been previously validated in pharmacy and dental student populations, their use in osteopathic medical education has not yet undergone formal validation. Future work should include psychometric analyses, such as confirmatory factor analysis and test-retest reliability, to ensure their applicability and accuracy in osteopathic cohorts.

## Appendices

**Table 4:** Survey instrument items Items from the Academic Entitlement Scale by Chowning and Campbell [7] are in the public domain and reproduced with attribution. Select items from the Academic Entitlement Questionnaire were adapted from Kopp et al. [8] and reproduced with permission from SAGE Publications.

Section	Question	Response options	Source
Demographics	What is your graduating class?	2027, 2026, 2025, 2024	Researcher-developed
	What age range do you currently fall within?	18-25, 26-40, 40 or more	
	What is your gender identity?	Male, female, non-binary, transgender female, transgender male, gender nonconforming, prefer not to answer	
Academic performance	How many reassessments did you complete in OMS-1?	1, 2, 3, or more	Researcher-developed
	How many reassessments did you complete in OMS-2?	1, 2, 3, or more	
	How many reassessments did you complete in OMS-3?	1, 2, 3, or more	
	Did you complete a course remediation during OMS-1?	Yes, no	
	Did you complete a course remediation during OMS-2?	Yes, no	
	Did you complete a course remediation during OMS-3?	Yes, no	
	What is your current class rank?	___ out of ___	
	What is your current GPA based on class average out of 100?	Open-ended	
Entitlement Instrument A	My professors are obligated to help me prepare for exams.	Likert scale (1-5)	Chowning and Campbell [7] Academic Entitlement Scale
	Professors are employees who get money for teaching.	Likert scale (1-5)	
	Professors must be entertaining to be good.	Likert scale (1-5)	
	I should never receive a zero on an assignment that I turned in.	Likert scale (1-5)	
	My professors should curve my grade if I am close to the next letter grade.	Likert scale (1-5)	
	I believe that the university does not provide me with the resources I need to succeed in college.	Likert scale (1-5)	
	For group work, I should receive the same grade as the other group members regardless of my level of effort.	Likert scale (1-5)	
	My professors should reconsider my grade if I am close to the grade I want.	Likert scale (1-5)	
	I believe that it is my responsibility to seek out the resources to succeed in college. (Reverse scored)	Likert scale (1-5)	
	Most professors do not really know what they are talking about.	Likert scale (1-5)	
	It is unnecessary for me to participate in class when the professor is paid for teaching, not for asking questions.	Likert scale (1-5)	
	I am not motivated to put a lot of effort into group work, because another group member will end up doing it.	Likert scale (1-5)	
	If I do poorly in a course and I could not make my professor's office hours, the fault lies with my professor.	Likert scale (1-5)	
	For group assignments, it is acceptable to take a back seat and let others do most of the work if I am busy.	Likert scale (1-5)	
	If I miss class, it is my responsibility to get the notes. (Reverse scored)	Likert scale (1-5)	
Entitlement Instrument B	If I am struggling in a class, the professor should approach me and offer to help.	Likert scale (1-5)	Kopp et al. [8] Academic Entitlement Questionnaire
	Professors should only lecture on material covered in the textbook and assigned readings.	Likert scale (1-5)	
	I should be given the opportunity to make up a test, regardless of the reason for the absence.	Likert scale (1-5)	
	If I don't do well on a test, the professor should make tests easier or curve grades.	Likert scale (1-5)	
	It is the professor's responsibility to make it easy for me to succeed.	Likert scale (1-5)	
	If I cannot learn the material for a class from lecture alone, then it is the professor's fault when I fail the test.	Likert scale (1-5)	
	I am a product of my environment. Therefore, if I do poorly in class, it is not my fault.	Likert scale (1-5)	
	Because I pay tuition, I deserve passing grades.	Likert scale (1-5)	
